# Assessment of Melt Compounding with Zeolites as an Effective Deodorization Strategy for Mixed Plastic Wastes and Comparison with Degassing

**DOI:** 10.3390/polym15081858

**Published:** 2023-04-13

**Authors:** Emilia Garofalo, Leonardo Taurino, Luciano Di Maio, Heinz C. Neitzert, Loredana Incarnato

**Affiliations:** Department of Industrial Engineering, University of Salerno, 84084 Fisciano, Italy

**Keywords:** recycled plastics, VOCs, off-odors, degassing, zeolite, sensory analysis

## Abstract

The emission of off-odors from mechanically recycled plastics severely limits their re-introduction into the market for the production of new objects, for the same use or even for less demanding applications, thus hindering the implementation of an effective circular economy for plastics. The addition of adsorbing agents during the extrusion of polymers represents one of the most promising strategy to reduce the odorous emissions of plastics, due to its characteristics of cost-effectiveness, flexibility and low energy consumption. The novelty of this work lies in the assessment of zeolites as VOC adsorbents during the extrusion of recycled plastics. They appear more suitable than other types of adsorbents, due to their ability to capture and “hold” the adsorbed substances at the high temperatures of the extrusion process. Moreover, the effectiveness of this deodorization strategy was compared with the traditional degassing technique. Two types of mixed polyolefin wastes, coming from completely different collection and recycling processes, were tested: Fil-S (Film-Small), deriving from post-consumer flexible films of small size, and PW (pulper waste), which is the residual plastic waste obtained from the paper recycling process. The melt compounding of the recycled materials with two micrometric zeolites (zeolite 13X and Z310) resulted as more effective in the off-odors removal with respect to degassing. In particular, the highest reduction (−45%) of the Average Odor Intensity (AOI) was measured for both PW/Z310 and Fil-S/13X systems at 4 wt% of the zeolites’ amount, compared with the corresponding untreated recyclates. Finally, by combining degassing and melt compounding with zeolites, the best result was obtained for the composite Fil-S/13X, whose Average Odor Intensity resulted as quite close (+22%) to the one of the virgin LDPE.

## 1. Introduction

A European survey on the use of recycled plastics materials (rPM), which was conducted in 2019 with the participation of 376 companies from 21 different countries, showed that the main reason preventing the plastics converting industry from using more rPM is the poor quality of these materials. In particular, among the main quality issues, there is the unpleasant odor of rPM [1,2]. Therefore, the establishment of methods for identifying the compounds responsible for off-odors in recycled plastics and the implementation of efficient and effective techniques to remove such substances play a key role to shift from a linear to a closed-loop plastic production system.

The odor perceived in most plastic materials (both virgin and recycled) is caused by the presence of volatile organic compounds (VOCs) which, according to the World Health Organization, can be classified into three categories by their volatility: very volatile organic compounds (VVOCs), with a boiling point lower than 100 °C; volatile organic compounds (VOCs), with a boiling point between 100 and 260 °C; and semi-volatile organic compounds (SVOCs), whose boiling point is between 260 and 400 °C [3,4]. A recent study [4] showed that most of the volatile substances inside recycled polymers can be included in the groups of VOCs and SVOCs. As a consequence, these compounds are difficult to devolatilize and could remain inside the material after manufacturing, thus lowering the quality of the final product. Under the category of VOCs fall several chemical substances: hydrocarbons, aromatic compounds, flavor and fragrance compounds, alcohols, aldehydes, carboxylic acids, ketones, esters, ethers, amines, amides, sulfur, and halogenated compounds [4,5,6,7].

VOCs can be generated by thermal degradation phenomena during polymer processing, which is the main reason for the presence of volatile substances in virgin polymers, and/or they can migrate into plastics at any other stage of their lifecycle including their production, storage, handling, packaging and disposal [8]. Thus, post-consumer polymers are usually more polluted and emit a wide variety of VOCs.

It is important to emphasize that not all the VOCs are responsible for the perception of specific smells that are strictly dependent on both the concentration and chemical composition of the substances causing them [4]. Moreover, since recycled plastics emit a large variety of VOCs, it’s not easy to isolate and analyze only the odor-active components. In this regard, to perform comprehensive smell characterization, instrumental methods, such as the most used Gas Chromatography-Mass Spectrometry (GC-MS), need to be combined with sensory analysis (GC-Olfactometry/MS) [3,4,5,6,7,9,10,11,12]. The sensory analysis, or panel test, allows us to determine the intensity and/or the odors’ characteristics by means of a group of evaluators, trained or not, depending on the procedure applied. In particular, since the smell perception is subjective, in terms of both intensity and pleasantness definition, some International (ISO), European (EN) or American (ASTM) standards were defined for the characterization of the odors emitted by solid matrices, with the aim of imposing common criteria and adding objectivity to the smells’ evaluation [4,10]. However, common regulations for the sensory analysis of odor-active compounds, specifically emitted from recycled plastics, have not been established to date [4].

Based on some recent studies, 437 VOCs have been overall reported until now, 80 of which were identified as odor-active substances in different sources of post-consumer plastic wastes, such as HDPE [6,9], LDPE, and PP resins [5], LDPE bags [11] and polyolefins from mixed film fractions [7]. Different deodorization techniques, applied to plastic wastes, were also reported in the literature [9,12,13,14,15,16,17,18]. In particular, degassing represents a common methodology, already used at an industrial scale for the processing of both virgin and recycled polymers. This technique is based on two main transport mechanisms: diffusion and bubble formation [13]. In particular, among the process controlling parameters, it is worth considering the interfacial area between the polymer melt and the vapor phase, as well as the residence time inside the extruder and the eventual addition of a stripping agent (water, CO_2_, etc.). This latter reduces the polymer viscosity, thus improving the VOCs’ diffusivity, and determines the generation of additional bubbles, with an increase in the melt/vapor interfacial area [13].

An innovative strategy, tested at a pilot scale for removing unwanted substances in post-consumer plastics, is based on the Extruclean technology [18]. It uses supercritical carbon dioxide (SC-CO_2_) as a stripping agent during the extrusion process to enhance the removal of contaminants by degasification. However, although the SC-CO_2_ extraction process seems effective to remove VOCs from plastics, it demands high energy consumption and requires a specific apparatus to bear the supercritical conditions.

Another very promising deodorization technique concerns the adsorption of VOCs by materials with a porous structure [19,20,21,22,23,24,25,26,27,28]. The main parameters that control this process are the adsorbent’s specific surface area, the pore structure and the adsorbent/adsorbate affinity. Moreover, it is worth pointing out that this technique works according to a selective adsorption mechanism, based on the ratio between the dimensions of the adsorbent’s pores and VOC molecules [19]. 

The effectiveness of several porous adsorbents, such as active charcoal as well as zeolite, towards many volatile compounds, had been widely demonstrated in the cases of fluid streams flowing through beds made of these adsorbents’ particles [19,21,22,23,24,25,27,28,29]. On the contrary, the assessment of the zeolites’ effectiveness as VOC adsorbents during the polymer extrusion process [16,26,27,30,31], thus at completely different boundary conditions (high temperatures and short contact times), needs more extensive investigations.

The objective of this study was to test two different approaches to remove volatile organic compounds, specifically the ones responsible for unpleasant odors, from two types of post-consumer polyolefin-based recycled materials: Fil-S (film-small), derived from flexible film plastic waste, and PW (pulper waste), which is the residual plastic waste obtained from the paper recycling process. 

The implemented strategies concerned the optimization of degasification conditions during the materials’ extrusion in a twin-screw extruder and, alternatively, the melt compounding of the recycled resins with porous adsorbents. In particular, the deodorization’s degassing technique effectiveness was evaluated by setting different residence times of the recycled plastics inside the extruder and by using water as a stripping agent. Concerning the other deodorization strategy, two kinds of micrometric zeolites were selected and VOC emissions from PW/zeolite and Fil-S/zeolite composites at different additive concentrations were assessed. Finally, these deodorization techniques were simultaneously implemented and the smell features of the treated recycled plastics were compared with the ones of virgin benchmarks.

The smell characterization of the recycled plastics, before and after each deodorization treatment, was conducted by both panel tests, performed by non-trained volunteers, and by using an electronic sensor. The combined use of these two methods for VOCs detection allowed us to obtain a quick and also reliable feedback, since these measurement techniques show complementary strengths and weaknesses. In particular, even if the perceptions of non-trained panelists may be subjective, they represent the average smell impression of the consumers. Conversely, the results obtained with a sensor are absolutely objective, but an electronic tool as powerful as the human nose does not currently exist. Moreover, one sensor alone is unable to discriminate between odor-active and odorless compounds.

## 2. Materials and Methods

### 2.1. Materials

Two kinds of recycled materials were used: Fil-S (film-small) and pulper waste (PW). The former represents a fraction of mixed plastics obtained by the sorting and mechanical recycling of post-consumer packaging films of small size (<0.125 m^2^) and was provided by COREPLA (the Italian National Consortium for the Collection and Recycling of Plastic Packaging), while the latter is the residual plastic waste obtained from the paper recycling process and was provided by the company Repulp s.r.l. (Lucca, Italy). 

A low-density polyethylene (Riblene FL30, supplied by Versalis S.p.A., San Donato Milanese, Italy) was used as a virgin polymer benchmark for comparison with the recycled resins in terms of odorous emissions.

Two types of micrometric zeolites were selected as porous adsorbents: the zeolite 13X (produced by Honeywell Fluka and purchased by Exacta-Optech, San Prospero, Italy) and the Zeoflair Z310 (by Zeochem, Louiswille, KY, USA). Before use, both zeolites were regenerated in a muffle furnace at 300 °C for 16 h.

### 2.2. Extrusion with Degassing and Melt Compounding with Zeolites

Degasification was carried out in a twin-screw extruder (Dr. Collin GmbH—model ZK 25-48D, Munich, Germany) equipped with co-rotating, intermeshing screws (L/D = 42 and D_screw_ = 25 mm) and a vacuum ventilation zone in the metering section, which allowed us to set a depression of −1 bar. The temperature profile and the screw speed, set for both the recycled resins, were 190-190-190-200-200-200-200-190 °C (from hopper to die) and 100 rpm, respectively. In order to study the effect of the residence time inside the extruder on the materials’ deodorization effectiveness, both the recycled resins were produced at two different flow rates (25 and 50 g/min) by properly dosing the pellets’ feeding through a gravimetric device. Furthermore, for each type of recyclate, one more sample at high residence time was produced, after conditioning them at 90% RH for 7 days.

The twin-screw extruder, at the same processing conditions reported above, but without degassing, was also used to produce masterbatches with the recycled materials at 20 wt% of the selected zeolites (PW+20% Z310 and Fil-S+20% 13X), so to effectively disperse the additives inside the recycled matrices. These masterbatches were, subsequently, diluted with the corresponding recycled polymer in a single-screw extruder (Brabender Do-Corder 330 with L/D = 20 mm and D_screw_ = 20 mm) to obtain samples (in form of ribbons) at different zeolite concentrations (2%, 4% and 6%wt) for each recycled resin. In particular, a screw speed of 20 rpm and a temperature profile of 155-155-155 °C for PW and of 165-170-160 °C for Fil-S were used, respectively. In order to evaluate how the adsorption competition between water molecules and VOCs affects the deodorization effectiveness of zeolites, polymer/zeolite composites were prepared by both pre-drying (at 70 °C under vacuum for 15 h) or not the recycled plastics before the processing by means of the single screw extruder. 

All the extruded samples were immediately stored in aluminum bags under vacuum with the purpose of preserving their odorous characteristics.

**Figure 1:**
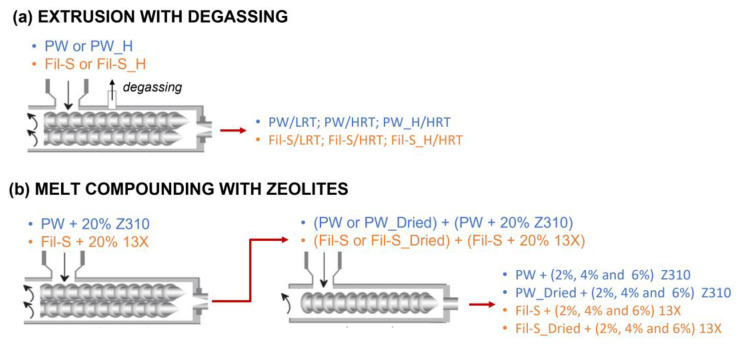
Schemes of the tested deodorization strategies: (**a**) extrusion with degassing and (**b**) melt compounding with zeolites.

For clarity, the nomenclature and description of all the samples are summarized in Table 1, while the schemes for both the tested deodorization strategies are reported in Figure 1.

### 2.3. VOCs/Odor Analysis

Based on the standard ASTM E619-84 [4], different groups of samples were analyzed by each panelist. The specimens were put inside obscured glass jars of fixed volume (106 mL), identified by a letter (A, B, C, D), so that the specimens could not be recognized by their name or appearance. A constant amount of material was introduced in each jar, so that each sample showed the same surface area (680 ± 10 mm^2^). All the specimens were pre-heated at 40 °C for an hour before the beginning of the panel tests, which were carried out by ten, non-trained volunteers. Each of the panelists was asked to fill in an online survey where he/she had to give a score from 1 to 5 to each sample, based on increasing odor intensity and he/she also had to give a short description of the perceived odors (Table 2).

Panelists’ answers were collected and to each sample was given an overall score, called Average Odor Intensity (AOI), obtained by using a weighted average of all the assigned scores:

(1) $$\bar{AOI}=\frac{\sum_{j=1}^{5}\left(I_{j}\times N_{j}\right)}{N_{tot}}$$

where “*I_j_*” is the odor intensity score relative to *j*-th sample; “*N_j_*” is the number of panelists who has given the intensity of “*I_j_*” to the *j*-th sample; and “*N_tot_*” is the total number of volunteers. 

After the panel test, all the jars were thoroughly washed, using a standard procedure.

The overall concentration of VOCs, emitted by each sample, was determined by using the electric sensor BME688, mounted on breakout I2C board (by BOSCH) and controlled by a Raspberry Pi2 microprocessor. The BME688 is a metal oxide-based sensor that detects gases by adsorption (and subsequent oxidation/reduction) on its sensitive layer, which results in an electrical resistance change of the thin film material. The sensor reacts to most volatile compounds, as well as many other gases polluting indoor air. In contrast to sensors selective for one specific compound, the BME688 can measure the sum of VOCs/contaminants in the surrounding air. The raw values are transformed into an Index for Air Quality (IAQ) by an appropriate algorithm. The IAQ scale ranges from 0 (clean air) to 500 (heavily polluted air). Measurements were carried out by following a three steps standard procedure:

- Start-up: sensor was turned on and left for calibration for at least 2–3 h in ambient air.
- Contact: the sensor was exposed to the material emitting VOCs, in the same configuration used for the panel tests: each sample with a constant surface area of 680 ± 10 mm^2^ was placed in a glass jar with a fixed volume (106 mL) and closed by a metallic lid, underneath which the sensor was mounted. This allowed to fix the measurement volume and the distance sensor-sample. Measurements were conducted for a time (20–30 min) long enough for the IAQ to reach a plateau value.
- Regeneration: the sensor was exposed to ambient air, allowing previously adsorbed VOCs to be released from the sensor’s surface. The sensor needed about 20 min to reach the initial condition and, therefore, to be considered as regenerated.

The collected data were shown in the following as bar graphs and the equilibrium value, reached by IAQ during the contact step, was considered as the reference value.

### 2.4. Characterization Techniques

The thermogravimetric analysis (TGA) was carried out using a Q500 analyzer (TA Instruments, New Castle, DE, USA), at a thermal scan rate of 10 °C/min in the range of 25–600 °C and under nitrogen atmosphere.

FTIR measurements were conducted in ATR mode on the neat materials in the range of 4000–650 cm^−1^, using a Nexus ThermoNicolet spectrometer (Thermo Scientific, Waltham, MA, USA). The spectra were collected with a resolution of 2 cm^−1^, co-adding 64 scans.

The scanning electron microscopy (SEM) analysis was carried out by means of a Zeiss EVO MA10 microscope (Carl Zeiss SMT AG, München-Hallbergmoos, Germany). In order to capture the images, the samples were sputter-coated with a 200–440 Å thick gold layer, using a Leica EMSCD005 metallization device.

Differential Scanning Calorimetry (DSC) analyses were carried out using a DSC30 Mettler calorimeter (Mettler-Toledo International Inc., Columbus, OH, USA) and performing the following thermal cycle: a first heating at 10 °C/min from 0 °C to 250 °C; an isotherm at 250 °C for 5 min to melt the residual crystals and remove the thermo-mechanical history; a cooling to 0 °C and a re-heating to 250 °C at the same scan rate.

## 3. Results and Discussion

### 3.1. Main Characteristics of the Recycled Plastics and Zeolites 

The physical–chemical characterization (DSC, FTIR, etc.) of Fil-S was already extensively conducted in our previous papers [30,32,33,34], evidencing that its main polymer components are polyethylene (LLDPE and LDPE) and a minor fraction of polypropylene (5%wt) with traces of polar contaminants.

**Figure 2:**
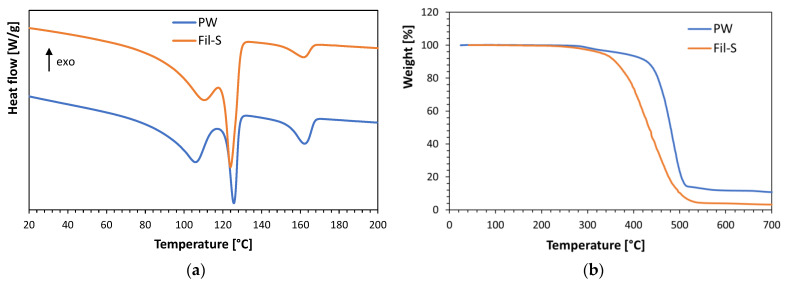
Comparison of the thermal data for PW and Fil-S: (**a**) 2nd heating DSC thermograms; and (**b**) thermogravimetric plots.

In Figure 2a,b, the second heating thermograms and the thermogravimetric plots of both the recycled materials are compared, respectively.

From the DSC curves (Figure 2a), it can be deduced that even though PW comes from a completely different waste collection and recycling process than Fil-S, they show a similar composition. In fact, PW is also mainly constituted by PE, but with a higher amount of PP (20%wt). Moreover, further information about the composition of PW can be obtained from the TGA results reported in Figure 2b. In particular, the thermogravimetric plot of PW shows a first weight loss of about 4.5% at c.a. 300 °C, which can be attributed to the breaking of the structural units of cellulose, thus evidencing the presence of a cellulosic residue inside this plastic waste. By further increasing the temperature, the most significant weight losses, equal to 97% for Fil-S and 85% for PW, are due to the degradation of the polyolefin fraction of both the recycled materials. Finally, an inorganic residue, equal to about 10% for PW and 3% for Fil-S, can be detected at the maximum temperature reached during the test (700 °C).

Regarding the selection of the zeolites, to be effective as adsorbents it is essential to consider their chemical affinity with the substances that should be adsorbed [19,20,21,22,23,24,25,27,28,29,33], as well as the dimensions of these latter with respect to the zeolites’ pore size. In particular, it was reported in the literature [19] that excellent adsorbent performances can be obtained if the ratio between the zeolite pore size (D_PORE_) and the kinetic molecular diameter of VOCs (D_VOC_) is in the range 1.7 < D_PORE_/D_VOC_ < 3. 

From the extensive characterization of the volatile compounds, conducted on plastic wastes with composition similar to PW and Fil-S [7,11,17], it resulted that the embedded odor-active substances show both a polar and non-polar character and have a molecular diameter between 2 and 8.5 Å [4,7]. Based on this information, zeolite 13X was chosen since its pore size (8.1 Å) should be adequate to adsorb most of the detected odorous compounds. The other porous additive, Zeoflair Z310, was selected since it is commercialized as a highly active zeolite-based adsorbent for the removal of undesirable odors and VOCs from adhesive, coatings, elastomers, etc. Its pore size is not disclosed by the manufacturer, but it is classified as having intermediate hydrophilic/hydrophobic properties [35], while the zeolite 13X has a predominant hydrophilic character (Si/Al ≅ 1.43) [36].

**Figure 3:**
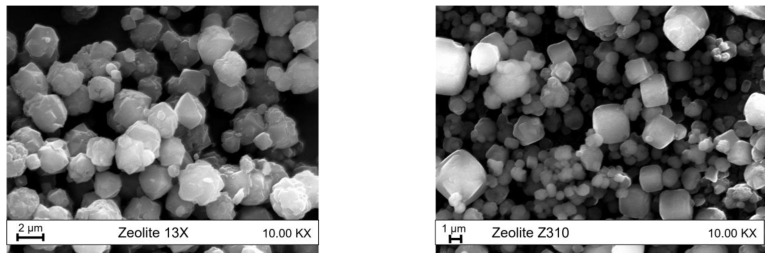
SEM images of zeolite 13X and Z310.

Both the selected zeolites were purchased as powders and, from the SEM images, reported in Figure 3, it can be deduced that they have similar particles’ dimensions (<10 µm). In particular, the granulometric distribution of zeolite 13X appears narrower than the one of Z310. Moreover, while the particles of this latter have an approximately cuboid form, zeolite 13X particles show a more complex geometric form.

**Figure 4:**
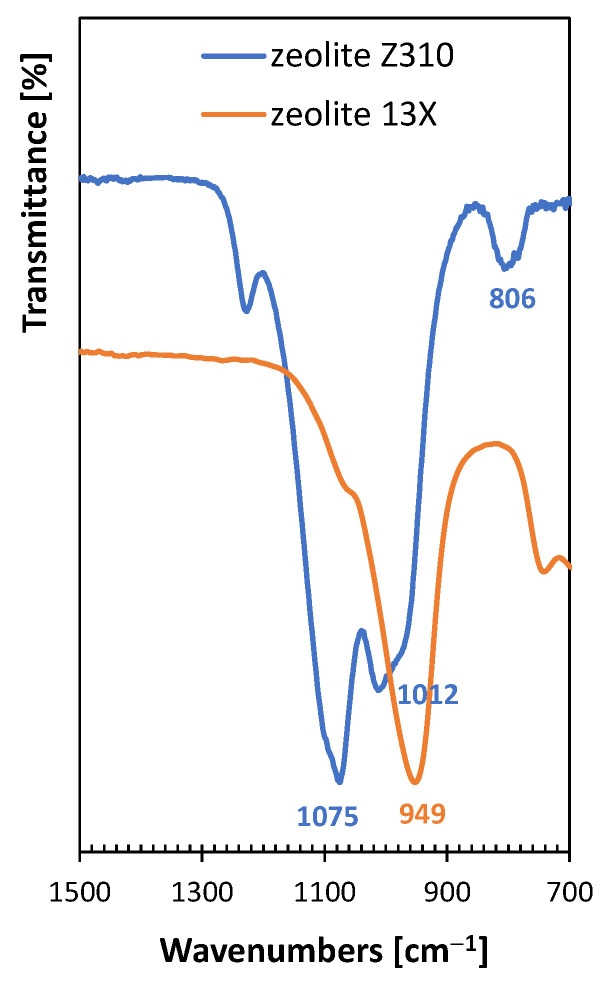
Comparison of FTIR/ATR spectra of the regenerated zeolites 13X and Z310.

FTIR/ATR analysis was also conducted on both the regenerated zeolites, and the resulting spectra are compared in Figure 4.

The zeolite 13X shows one well-defined band at 949 cm^−1^, characteristic of the alumino-silicate groups that constitute the building structure of zeolites [37]. A more complex peak can be observed for zeolite Z310. In particular, the band at 806 cm^−1^ belongs to the vibrations of the Si-O bond and the intense bands in the 900–1100 cm^−1^ region correspond to the stretching vibrations of the Si-O bond (1075 cm^−1^) and Al-O bond (1012 cm^−1^) in the mixed silicon–aluminum–oxygen skeleton [38]. 

### 3.2. Extrusion with Degassing: Optimization of the Process Conditions

As reported in the Experimental section (Section 2.2), both the recycled plastics were processed with degassing at two different residence times: LRT = 40 s and HRT = 120 s. Moreover, samples with higher water content (PW_H and Fil-S_H) were also extruded with the purpose of evaluating the effect of water as a stripping agent on the deodorization effectiveness of degassing.

**Figure 5:**
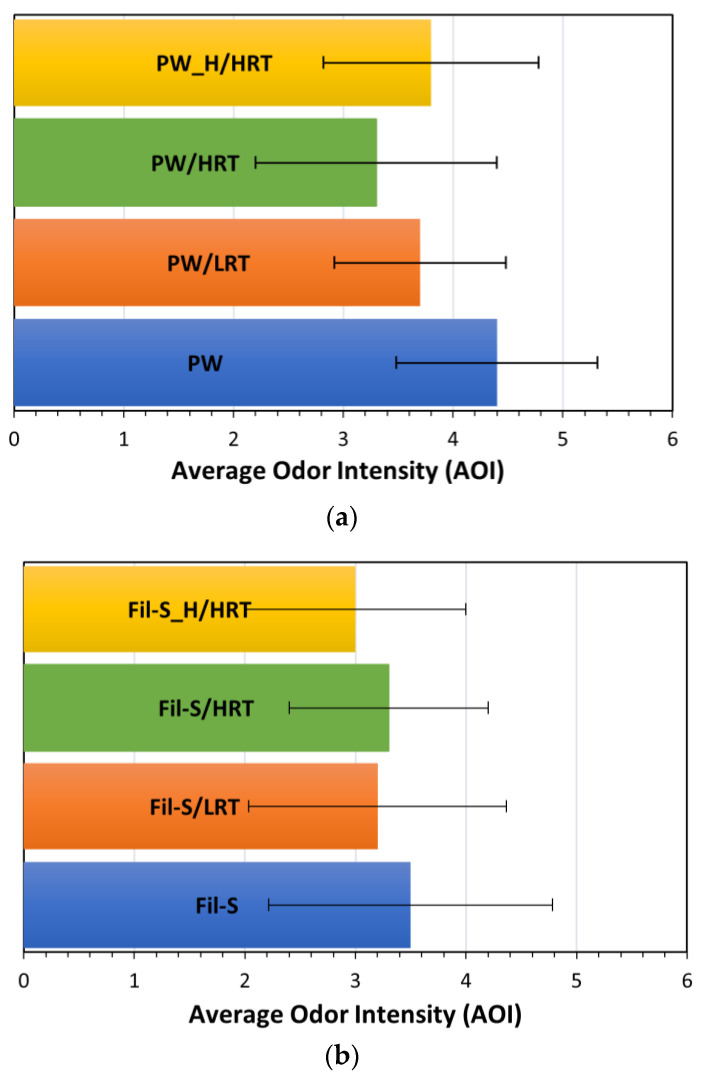
Panel tests’ results on: (**a**) PW and (**b**) Fil-S samples subjected to different degassing conditions.

In Figure 5a,b, the panel tests’ results are reported in terms of Average Odor Intensity (AOI) for the specimens based on PW and Fil-S, respectively.

The graphs show that the volunteers judged the odor emitted from the degassed samples slightly less intensely than the untreated ones, even if the AOI values are relatively close to each other. Moreover, degasification resulted as less effective on the deodorization of Fil-S compared to PW. Specifically, PW/HRT evidenced the lowest AOI value, with a reduction of 25% compared to the untreated PW, while in the case of Fil-S, the humid specimen Fil-S_H/HRT showed the greatest reduction in odor intensity with respect to the untreated Fil-S. The higher moisture content of Fil-S_H compared to PW_H (Table 1) suggests the need to reach a threshold value in the water amount in order to be effective as a stripping agent.

**Figure 6:**
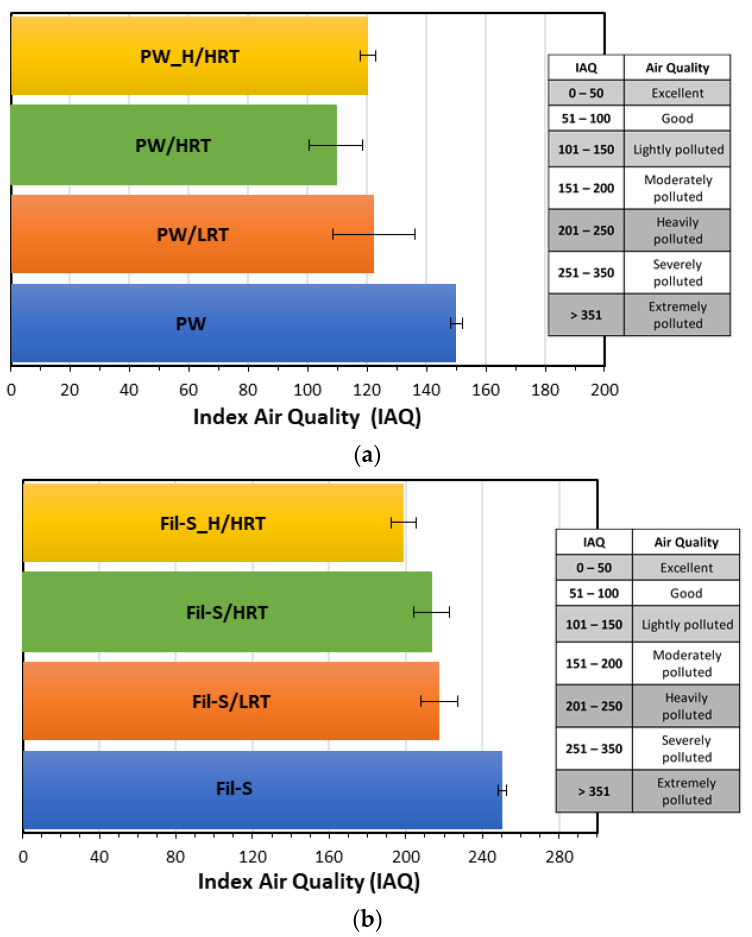
Index for Air Quality (IAQ) values for: (**a**) PW and (**b**) Fil-S samples subjected to different degassing conditions.

All the samples were also analyzed with the sensor. As specified in detail in the Experimental section (Section 2.3), this sensor allows us to obtain an Index for Air Quality (IAQ), which is correlated with the total level of VOCs (both the odor-active substances and the odorless ones) present in the environment where the material is confined. The results of the analysis with the sensor are reported in Figure 6a,b for PW- and Fil-S-based specimens, respectively.

The IAQ results were consistent with the trends observed from the panel tests, with PW/HRT and Fil-S_H/HRT samples showing the lowest values of IAQ (−27% and −20%) compared to the untreated PW and Fil-S, respectively. 

In other words, the measurements with the sensor confirm an interesting reduction of the total VOCs emitted by the materials subjected to degassing, even if this deodorization strategy is not able to significantly remove the volatile compounds responsible for the bad smell of these recycled plastics, as highlighted by the panel tests. In fact, according to the panelists’ opinion, the treated samples have Average Odor Intensity values quite close to the untreated ones, suggesting the need for alternative and/or complementary deodorization strategies to the degassing technique.

### 3.3. Melt Compounding with Zeolites

#### 3.3.1. Preliminary Tests for the Selection of Zeolite Type for Each Recycled Resin

In order to properly match the zeolite type with each recycled matrix, 30 g of Fil-S or PW were mechanically mixed with 2% by weight of each zeolite, obtaining the following dry blends: Fil-S+2% 13X, Fil-S+2% Z310, PW+2% 13X and PW+2% Z310.

**Figure 7:**
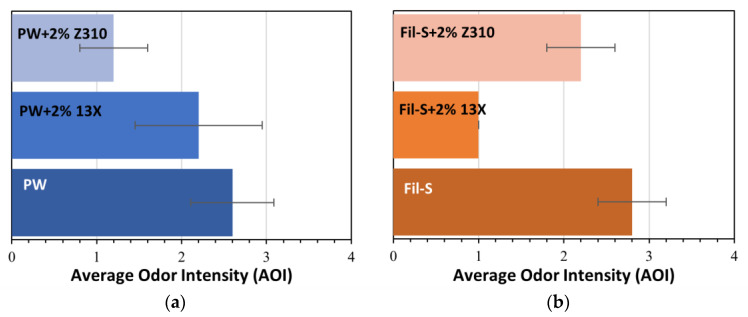
Results of the preliminary panel tests on (**a**) PW- and (**b**) Fil-S-based composite with the zeolites.

Two preliminary panel tests, for PW-based systems and Fil-S-based ones, were carried out. Three samples in obscured glass jars (106 mL) were proposed to each panelist: one with the pellets of the recycled resin as such and the other two containing the dry blends of the corresponding recycled material with the two different types of zeolite. The panelists were asked to rank each sample in ascending order of odor intensity, giving a score from 1 “less intense” to 3 “more intense”. Panelists’ answers were collected and an average score, based on a similar expression as AOI (Equation (1)), was given to each sample. The results of these preliminary panel tests for PW- and Fil-S-based systems are reported in Figure 7a,b, respectively.

In the case of PW, the blend with 2% of Z310 resulted the system with the least intensity of smell, while, regarding Fil-S, the best sample was Fil-S+2% 13X.

In order to objectively assess the VOC adsorption by both the selected zeolites, they were subjected to thermogravimetric analysis after they’d been in contact (as dry blends) with the recycled matrices for a week. The obtained curves were compared with the corresponding graphs of the regenerated zeolites. Both zeolites were also conditioned at high relative humidity (RH= 90%) until saturation and then were analyzed by TGA with the aim to verify any competition, in terms of adsorption kinetics, between VOCs and water vapor.

**Figure 8:**
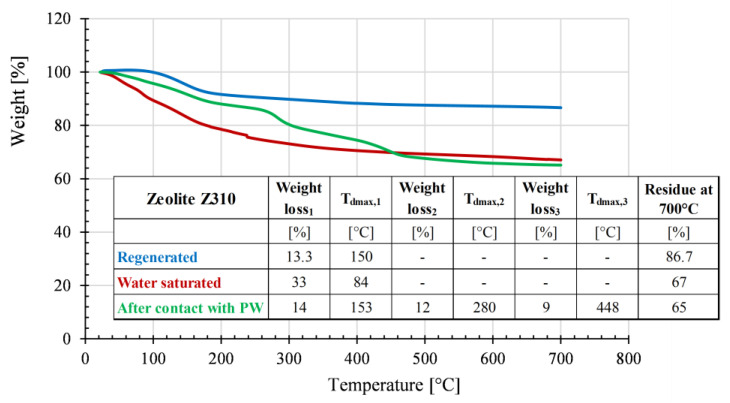
Results of the thermogravimetric analysis conducted on zeolite Z310 samples: after its regeneration in a muffle, water saturated and after being in contact with PW as dry blend.

**Figure 9:**
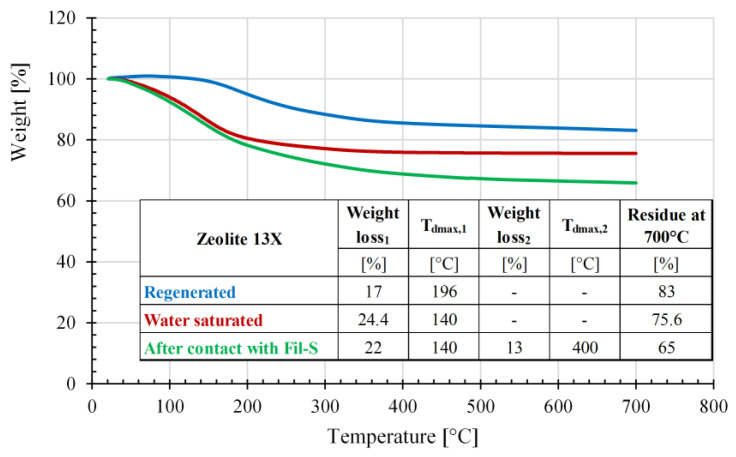
Results of the thermogravimetric analysis conducted on zeolite 13X samples: after its regeneration in a muffle, water saturated and after contact with Fil-S as dry blend.

The thermogravimetric curves for the zeolites Z310 and 13X and the corresponding details, in terms of degradation temperatures and weight losses, are reported in Figure 8 and Figure 9, respectively.

The regenerated Z310 shows only one weight loss of about 13% at 150 °C, probably due to the removal of strongly bounded water molecules. On the contrary, the water-saturated Z310 zeolite evidences a more pronounced weight loss (about 33%) that covers a wider temperature range. The TGA plot of Z310, after it has been in contact with PW, displays multiple weight losses at different temperatures, thus suggesting a selective adsorption mechanism: the first weight loss, of 14% at T = 153 °C, is similar to the one observed for the regenerated Z310, while the other losses (12% at 280 °C and 9% at 448 °C) are reasonably due to the desorption of VOCs with higher boiling points. To obtain a deeper insight about the identification of the adsorbates released by the zeolites, further investigations should be performed by coupling TGA analysis with mass spectrometry.

Regarding the TGA results on zeolite 13X, it was observed that the regenerated sample shows one weight loss of 17% at 196 °C, probably due, also in this case, to the removal of strongly bounded water molecules. The plots of the water saturated zeolite 13X and the one that has been in contact with Fil-S almost overlay in the temperature range 0–160 °C, evidencing a similar weight loss (of 24.4% and 22%, respectively) at 140 °C. This suggests a possible competition between the adsorption mechanisms of water molecules and VOCs. Finally, another weight loss of 13% at 400 °C was observed for the zeolite 13X after being in contact with Fil-S. This can be reasonably attributed to the removal of high boiling VOCs.

#### 3.3.2. Optimization of the Zeolites’ Concentration

After the selection of the more suitable type of zeolite for each recycled resin, the composites Fil-S/13X and PW/Z310 were prepared by melt compounding at different amounts (2, 4 and 6 wt%) of the adsorbent additive, using both the pre-dried (PW_Dried and Fil-S_Dried) and non-dried recyclates. 

**Figure 10:**
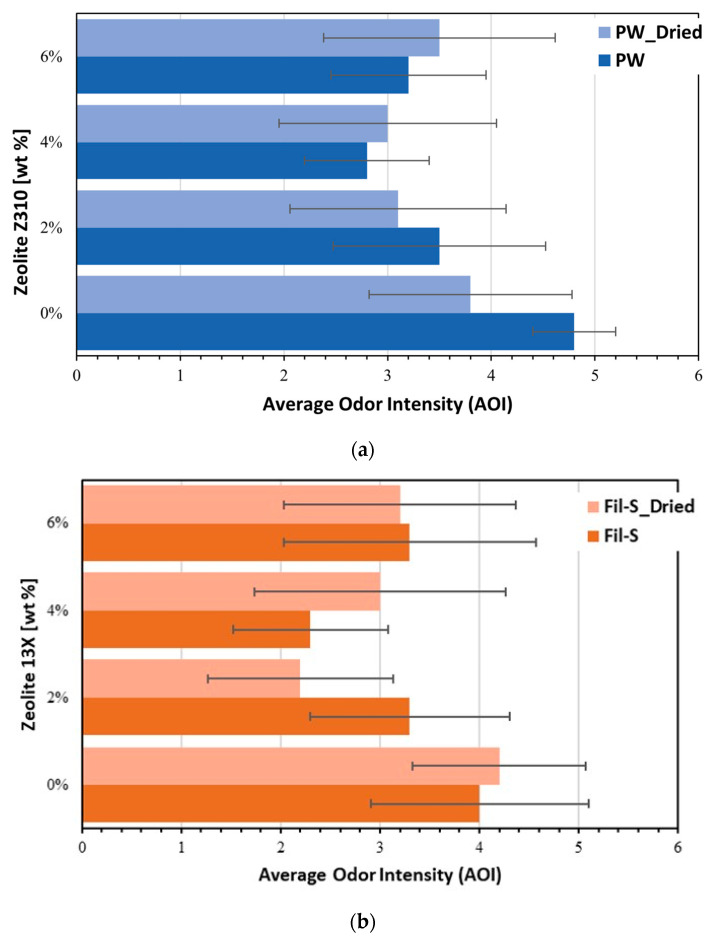
Panel tests’ results for: (**a**) pre-dried and non-dried PW with different concentrations of Z310; and (**b**) pre-dried and non-dried Fil-S with different concentrations of 13X.

In Figure 10a,b, the panel tests’ results for PW and Fil-S based composites are reported at increasing amounts of zeolite Z310 and 13X, respectively.

A reduction of the odor intensity (AOI) can be observed for all the composites compared with the corresponding recycled matrices. In particular, in the case of PW based systems, the parameter AOI decreases by increasing the concentration of zeolite reaching a minimum at 4% of Z310, which corresponds to a 42% of odor intensity reduction compared to the unfilled recycled material. At higher zeolite content (6 wt%) an increment of the odor intensity was perceived by the panelists. This result can be reasonably attributed to the aggregation of the zeolite, with consequent reduction of the available adsorbent’s specific surface area, which directly affects its adsorption capacity. 

**Figure 11:**
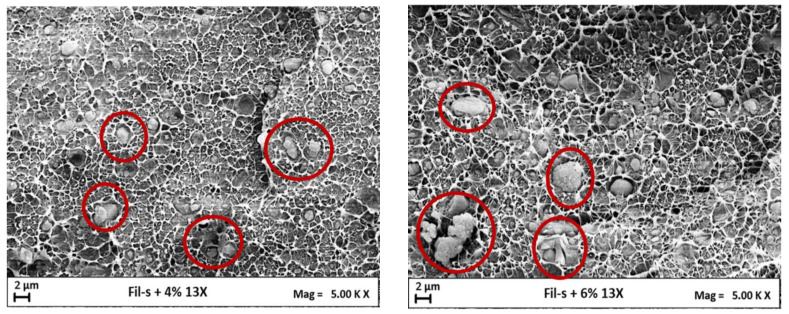
SEM images of Fil-S+4% 13X and Fil-S+6% 13X. Red circles highlight zeolite’s particles.

The same trend, as a function of the zeolite percentage, was also shown by Fil-S/13X systems (Figure 10b). Moreover, the hypothesis of the additive’s aggregation, at the highest content tested, was confirmed by SEM images of the composites Fil-S/13X reported in Figure 11. In fact, zeolite’s aggregates of bigger dimensions (as highlighted by the red circles) can be clearly observed inside the sample Fil-S+6% 13X with respect to the system at the lower additive’s concentration.

**Figure 12:**
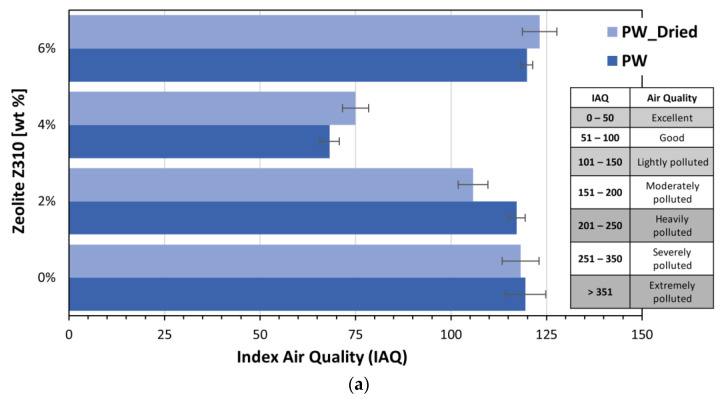

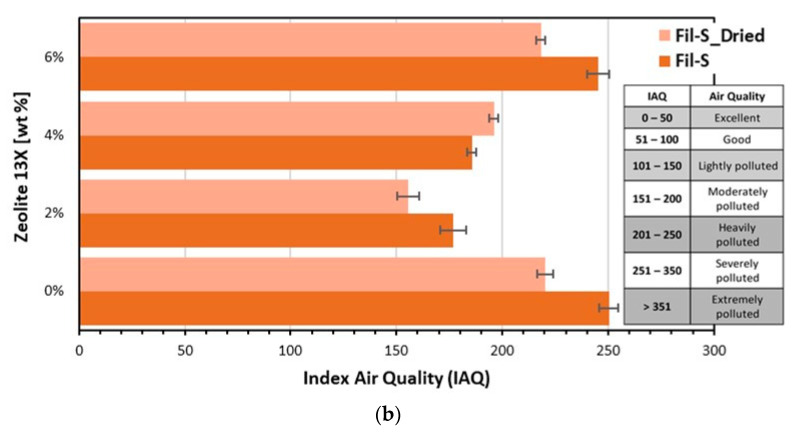
Index for Air Quality (IAQ) values for: (**a**) pre-dried and non-dried PW with different concentrations of Z310; and (**b**) pre-dried and non-dried Fil-S with different concentrations of 13X.

Comparing the AOI values for the dried and non-dried samples, in the case of PW-based systems (Figure 12a), a significant difference in the odor intensity was perceived only for the unfilled recycled material. In particular, the dried PW sample showed a lower bad smell intensity compared with the non-dried one.

For Fil-S based systems, the minimum value of AOI appeared at different percentages of 13X: at 2% for the dried samples and at 4% for the non-dried ones. This behavior suggests a possible competition between the adsorption mechanisms of water molecules and VOCs on the porous structure of zeolite 13X, in agreement with the TGA results (Figure 9). In other words, the non-dried specimen at 2% of zeolite 13X is able to adsorb less odor compounds compared with the dried sample because its active sites are probably partially filled by water molecules.

VOCs detection in all the recyclate/zeolite systems was also carried out using the sensor and the results obtained are summarized in Figure 12a,b.

In the case of PW-based composites, the lowest values of IAQ were measured at 4% of zeolite Z310 for both the dried and non-dried samples. 

For Fil-S-based systems, the minimum IAQ data were registered at 2% of zeolite 13X, even if, by increasing the zeolite content at 4%, the IAQ values were still included in the range corresponding to “moderately polluted” air, as for the specimens at lower zeolite amounts. 

In conclusion, the addition of 4% zeolite (Z310 for PW and 13X for Fil-S, respectively) inside the non-dried recycled materials allowed us to obtain a reduction of 42% in odor intensity with respect to the unfilled recyclates, significantly higher than the best deodorization results obtained by extruding with degassing the same recycled plastics (Section 3.2).

### 3.4. Combination of Degassing and Compounding with Zeolite

Finally, extrusion with degasification and zeolite compounding were combined. In particular, the best conditions obtained for both the techniques were used and the panel tests were performed on the following two sets of specimens:

- PW; PW/HRT+4% Z310; LDPE; LDPE+4%Z310;
- Fil-S; Fil-S_H/HRT+4%13X; LDPE; LDPE+4% 13X.

**Figure 13:**
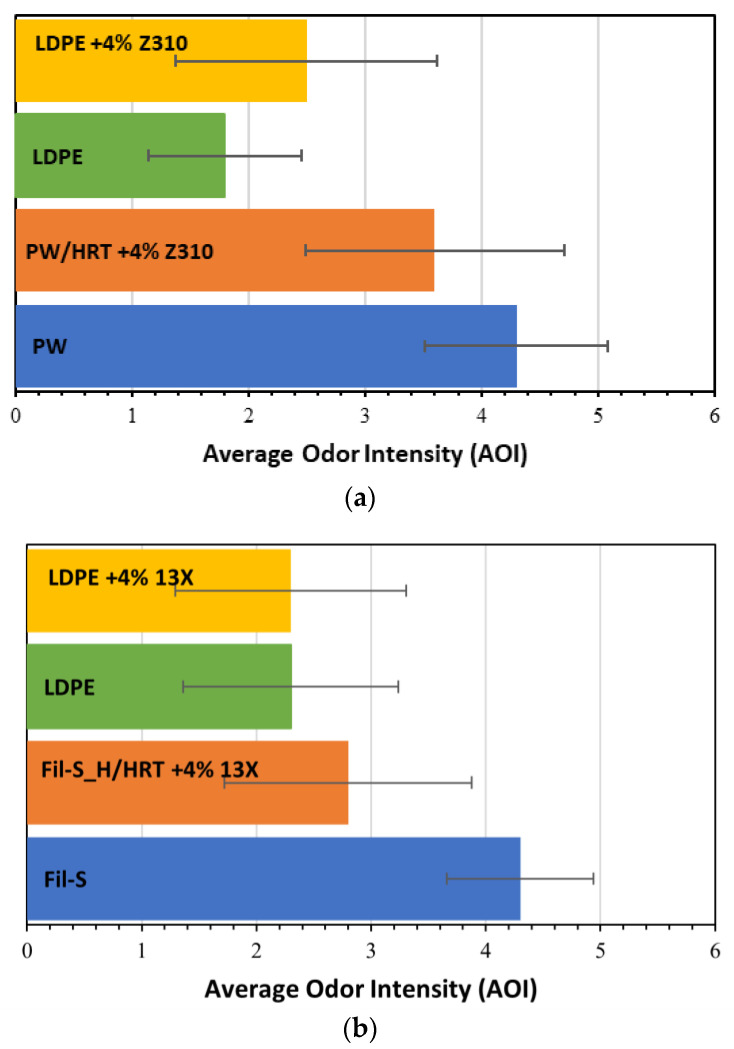
Panel tests’ results: (**a**) comparison of the treated sample PW/HRT+4% Z310 with PW as such, virgin LDPE and LDPE+4% Z310; and (**b**) comparison of the treated sample Fil-S_H/HRT+4% 13X with Fil-S as such, virgin LDPE and LDPE+4% 13X.

Virgin LDPE was chosen as a benchmark resin, since both Fil-S and PW are mainly constituted by polyethylene. Moreover, in order to consider the eventual odor emissions from the zeolites themselves, the treated recyclates were also compared with the LDPE/zeolite composites. The results on the smell perception for all these specimens are reported in Figure 13a,b.

**Figure 14:**
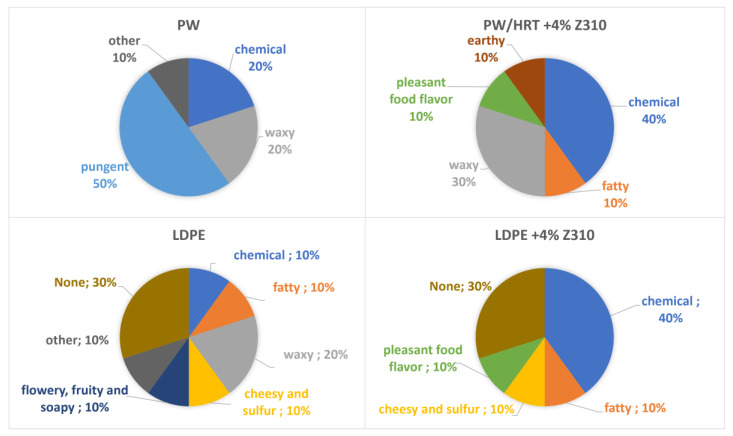
Description of odors perceived by the panelists about the samples: PW, PW/HRT+4% Z310, LDPE, LDPE+4% Z310.

From the data in Figure 13a, it can be deduced that even the neat LDPE shows a perceptible odor. Moreover, the addition of the zeolite Z310 in the virgin polymer (LDPE+4% Z310) significantly contributes in increasing its odor emissions. Regarding the recyclates, a discrete reduction (−16%) of the odor intensity was assessed by the panelists for the specimen PW/HRT+4% Z310 compared with the untreated PW, but its smell was judged significantly higher than the virgin LDPE. For a better understanding of these results, it is useful to consider how the panelists have described the perceived odors (Figure 14).

For most of the panelists, the sample PW/HRT+4% Z310 had a chemical smell, as in the case of the virgin polymer filled with the zeolite Z310, while half of them described the odor of PW as pungent. In other words, the smell perceived for the sample PW/HRT+4% Z310 can be partly attributed to the odor of the zeolite itself.

The panel test conducted on Fil-S-based samples and the virgin resins revealed a more significant reduction of AOI (−35%) for the treated recycled material compared to the untreated Fil-S. Moreover, LDPE and LDPE+4% 13X showed the same value of odor intensity, suggesting that zeolite 13X does not contribute to the odor emissions. In conclusion, the average odor intensity of the specimen Fil-S_H/HRT+4% 13X resulted as only 22% higher than LDPE.

## 4. Conclusions

One of the major challenges for upgrading mechanically recycled materials to make them fit to reuse for the same or new applications concerns the removal of malodorous substances embedded inside the polymers. This study focused on the deodorization of two types of plastic waste: PW and Fil-S. Even if they come from completely different waste collection and recycling processes, they show a quite similar composition, being constituted mainly of polyethylene and a minor content of polypropylene. Two deodorization techniques were implemented on these post-consumer mixed plastics, in particular a new promising strategy, i.e., the melt compounding of the recycled materials with zeolites, was compared with the more traditional extrusion with degassing, which is already applied at an industrial scale for VOC removal.

Based on the results obtained in this study, the degassing technology was not able to reduce, to a satisfactory extent, the unpleasant smells of both the recycled plastics, despite the optimization of the residence time inside the extruder and the use of water as a stripping agent.

The melt compounding of the recycled resins with the zeolites resulted as more effective than degassing in the reduction of bad smell. In particular, the lowest odor intensity (−45% with respect to the untreated recycled materials) was associated with the composites PW/Z310 and Fil-S/13X at 4% by the weight of both the zeolites. A further increase in the content of the additives promoted their aggregation, thus reducing the zeolites’ adsorption capacity. 

Finally, the odor features of the samples, obtained by combining degassing and compounding with zeolites at the best conditions found for both the techniques, were compared with a virgin resin. Even if the treated PW and Fil-S specimens still showed an average odor intensity higher than the benchmark, in the case of the sample Fil-S/13X, this value was quite close (+22%) to the one of the virgin resin. 

In conclusion, this study highlighted that the melt compounding of post-consumer-mixed polyolefin wastes with zeolites represents an effective, industrially feasible and inexpensive deodorization strategy, which could greatly favor the reintroduction of these recycled resins into the production cycle, according to a circular approach.

## Figures and Tables

**Table 1:** Samples’ nomenclature and description.

Samples Prior Processing	
Sample name	ppm H_2_O
PW	2914 ± 115
Fil-S	2265 ± 108
PW_Dried	558 ± 35
Fil-S_Dried	459 ± 44
PW_H	3372 ± 122
Fil-S_H	5701 ± 214
Samples after processing in twin-extruder	
Sample name	Description
PW/LRT	Low Residence Time = 40 s inside the extruder
Fil-S/LRT	
PW/HRT	High Residence Time = 120 s inside the extruder
Fil-S/HRT	
PW_H/HRT	Humid samples processed at HRT inside the extruder
Fil-S_H/HRT	

**Table 2:** Odor intensity rating scale and description of the perceived odors [4].

Odor Intensity	Numerical Rating
None	1
Very slight	2
Slight	3
Moderate	4
Strong	5
Type of odor	Description
Chemical	Solvent, alkane, aromatic, petrol, gasoline, plastic and phenolic odors
Fatty	Fatty, oily smell
Waxy	Wax-like smell
Cheesy and sulfur	Onion-like, garlic-like and cheesy smell
Pungent	Acidic, musk, spicy, fecal, pea-like, malty and rancid odors
Pleasant food flavor	Caramel-like, chocolate, butter-like, bready, coffee-like and vanilla odors
Flowery, fruity and soapy	Berry, balsamic, citrus, herbal and minty odors
Earthy-moldy	Wet earth, mold-like smell

## Data Availability

Data are contained within the article.
